# From Prompt to Drug: Toward Pharmaceutical Superintelligence

**DOI:** 10.1021/acscentsci.5c01473

**Published:** 2026-02-02

**Authors:** Alex Zhavoronkov, David Gennert, Jiye Shi

**Affiliations:** † 612614 Insilico Medicine US Inc, Cambridge, Massachussetts 02138, United States; ‡ Insilico Medicine AI Ltd, Masdar City, Abu Dhabi 145748, UAE; § Insilico Medicine Shanghai Ltd., Shanghai 201203, China; ∥ 1539 Eli Lilly and Company, San Diego, California 92121, United States

## Abstract

The convergence of generative artificial intelligence (AI) platforms and automated laboratory systems is ushering in a new era of drug discovery, in which a plain-language prompt can initiate a fully autonomous, end-to-end drug development program. This article explores the recent evolution of AI technologies and presents a “prompt-to-drug” pipeline, where AI not only generates novel hypotheses and designs optimized drug candidates but also orchestrates synthesis, validation, and clinical planning in a closed-loop system. By highlighting key breakthroughs, case studies, and the technological infrastructure required for this paradigm shift, we outline a vision for scalable, efficient, and unbiased drug discovery.

## Graphical abstract

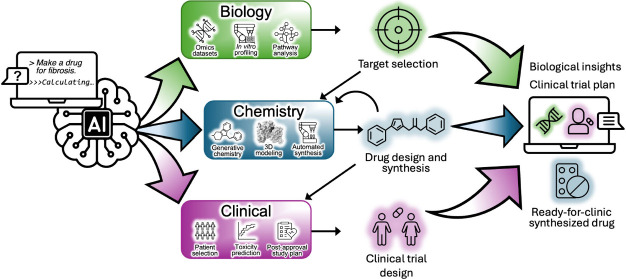

## 1 Introduction

The future of drug development, fueled largely by the past decade of monumental advances in artificial intelligence (AI) and the availability and integration of multiomics data sets into discovery pipelines, is coalescing around the idea of “prompt-to-drug”. The recent advance of AI-designed drugs into the clinic^1,2^ and rapid deployment of AI-based language models across industries has heralded a shift towards AI-accelerated drug discovery pipelines. Plain-language input and output from generative AI platforms and promising proofs-of-concept demonstrations of handing off complex computation and systems control to autonomous systems lead to a scenario in which a scientist could initiate an entire drug development program by simply describing the desired therapeutic outcome to an AI model. From a prompt alone, a fully integrated AI-driven system would autonomously identify relevant targets, design potent and safe compounds, guide synthesis, plan and execute preclinical studies, and even draft clinical trial protocols.

Unlike the traditional, siloed approach to pharmaceutical development, the prompt-to-drug model promises a seamless, adaptive, and highly efficient pipeline. Each stage, i.e., target identification, molecular design, biological validation, and clinical planning, is not only accelerated but also dynamically informed by feedback from preceding and ongoing experiments. Elimination of many of the bottlenecks caused by human limitations in data integration, experimental throughput, and hypothesis generation has the potential to dramatically reduce development timelines, cut costs, and increase the probability of success in clinical trials.

Here, we present a comprehensive overview of the technologies, milestones, and conceptual frameworks that underpin this transition toward fully autonomous drug development. We trace the evolution of AI in the pharmaceutical sciences, from rule-based systems and machine learning to the modern era of LLMs and multi-agent reasoning systems, exploring how these innovations are being assembled into end-to-end workflows.

## 2 Historical Evolution of AI in Drug Discovery

### 2.1 Traditional Machine Learning

The use of AI-based tools throughout the drug discovery process is not new, having been implemented in target discovery, medicinal chemistry/small-molecule design, and the design of biologics since the earliest opportunities (Figure 1). Traditional machine learning approaches are used extensively in general screening, classification, and relatively simple similarity-based screening.^3–6^ These algorithms are able to handle noisy biologic data, are often robust to overfitting, and commonly have high interpretability. ML methods can prognostically classify inhibitors for disease-associated molecular targets,^7^ predict and score drug-target interactions,^8,9^ and discover key properties of targets and candidate drugs, such as target druggability^10^ or pharmacophore specificity.

**Figure 1:**
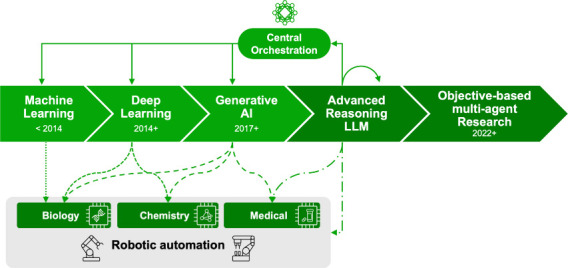
Evolution of AI in biotechnology. The advance of artificial intelligence algorithms and architecture over the past decade has expanded the reach of AI-based tools from omics analyses of biological systems into molecular design and medicinal chemistry and into clinical practice and clinical trial design. Automation of steps within scientific discovery and drug development processes controlled by the latest advanced reasoning algorithms fuel increased throughput and cross-disciplinary workflows.

### 2.2 The Deep Learning Revolution

Developed in the early 2000s and into the 2010s, deep learning (DL) algorithms leveraged advances in the massively parallel computational architecture of graphics processing units (GPUs) and were rapidly implemented in a wide array of fields including drug discovery. Molecular dynamics simulations especially rely on GPU-powered parallelization, which advanced at a rate beyond Moore’s Law,^11^ allowing for modelling of drug-target binding, docking simulations, and structure-activity studies for the design of drug molecules.^11^ Virtual screening with artificial neural networks in particular, taking advantage of high-dimensional multi-omics datasets, has been used to screen for peptides with antibiotic properties and predicting compound binding affinities.^12–15^

### 2.3 Advent of Generative AI

In the mid-late 2010s, advances in DL computation, notably the development of the variational autoencoder, generative adversarial networks (GANs), and the transformer model, led to an explosion of powerful and diverse generative AI-based tools (Figure 1). With each leap forward in AI capability, from ML to DL to generative AI, each successive modality has been able to take on additional phases in the biotechnology development cycle: ML analysis of omics data facilitated discovery of pathogenic mechanisms and disease targets,^16,17^ DL algorithms that built upon ML frameworks improved protein-protein,^18,19^ drug-target,^20,21^ and drug-drug^22–24^ interaction analyses with chemical and structural insights, and generative models further expanded molecular design capabilities with generative chemistry^25–29^ and clinical trial prediction^30–34^ functionality (Figure 1).

Initially, deep learning-based generative methods were restricted to 1D or 2D molecule outputs, as algorithms were classically trained on text-based molecular representations,^35^ like SMILES^36^ or SELFIES,^37^ or on graph representations of molecules.^38^ A variety of generative AI methodologies have since been developed to leverage the increasing availability and resolution of 3D structural data on potential target proteins,^39^ allowing models to be trained on 3D representations of molecules for more accurate and efficient prediction of intrapocket binding,^40^ from graph neural networks sequentially assembling atoms^41,42^ to diffusion-based full-molecule methods.^43,44^

The time- and money-saving potential of these generative models is exemplified by the generative tensorial reinforcement learning (GENTRL) model by Insilico Medicine that built upon the autoencoder models developed for learning molecular structure-informed properties,^25,26,45,46^ which discovered potent and selective inhibitors of DDR1 in only 21 days, followed by synthesis and validation in an additional 27,^29^ as well as a CDK20 inhibitor within 30 days.^47^

Large language models (LLMs) leveraged as foundation models, such as generative pretrained transformers (GPTs), have made powerful AI-based tools accessible and simple to implement in healthcare and drug discovery applications. BioGPT^48,49^ and commercially available platforms such as ChatGPT^50^ and PandaOmics^51–54^ can uncover biological networks and therapeutic targets, leveraging large-scale text sources, from research articles to patents to grants. Other GPT-based models can generate novel drug compound structures based on text-based SMILES or SELFIES representations of molecules,^55^ which lend themselves nicely to the token-pattern prediction capabilities of LLMs. cMolGPT,^56^ ChemGPT,^57,58^ DrugGPT,^59^ and MTMol-GPT^60^ are GPT-based generative chemistry models trained on large, publicly available datasets, such as ChEMBL and PubChem.

Inherent design features of today’s LLM tool landscape, however, make them suboptimal for end-to-end molecular discovery tasks. The pattern-recognition basis for generative LLMs lacks a deep understanding of the biochemical principles underlying chemical structure-dependent properties and complex interactions within biological systems.^61^ The tokenization of SMILES or SELFIES further simplifies chemical representations and loses information, such as stereochemical properties, that could inform docking models and other higher-order molecular properties.^55,61^ Multimodel approaches that either sequentially or iteratively call on different tools may be a solution to capture the benefits of each tool while reducing the impact of lost information or throughput inherent to each method.^62^ Generative models also have difficulty exploring chemical space outside their training sets, but specialized training to resolve this runs into issues of prohibitive computational, monetary, and time expenses involved in training and validation of LLMs.^55^

Moreover, the piecemeal development of AI tools designed to address individual steps along the drug discovery process has resulted in inefficiencies, creating downtime when switching between tools, users, or experts providing feedback. A system capable of smoothly handing off tasks will be key to resolving this inefficiency and allow a fully end-to-end pipeline to leverage all of the tools available (Figure 1).

## 3 Toward End-to-End AI-Accelerated Drug Discovery

### 3.1 Integrating Disparate Processes with an Intelligent System of Systems

The modular nature of existing AI-based tools facilitating drug development has arisen from efforts accelerating or automating each step in the process, such as target discovery or the SAR optimization of candidate drug molecules. The real revolution will come from linking these various systems into truly end-to-end pipelines. Improvement in development cycle time and reduction of the reliance on human coordination and data analysis at each step will be crucial for maximizing efficiency. Indeed, the very nature of these modular existing platforms will play a key role in enabling this hands-off approach, as AI algorithms are highly capable of integrating disparate data types and functionalities.^63^ Machine learning approaches with layered and network-based data integration of various data modalities such as omics, imaging, clinical, and text mining data sources have long been established as powerful tools for discovery of disease progression biomarkers,^64^ novel drug targets,^51^ and prediction of therapeutic response^65^ and survival.^66^

The modular nature of existing AI-based tools facilitating drug development has arisen from efforts accelerating or automating each step in the process, such as target discovery or the SAR optimization of candidate drug molecules. The real revolution will come from linking these various systems into truly end-to-end pipelines.

Integration of these drug discovery subsystems would benefit from a directed system-of-systems approach to orchestration, wherein independently operating component systems, such as target discovery, molecular design, and biological validation, are subordinated to a central controller.^67^ With drug discovery subsystems themselves relatively self-contained and already in the process of being optimized for automation, central management is most effective for the purpose of interfacing between systems.^67^ Intelligent system-of-systems, leveraging AI to improve performance, have been envisioned as process orchestrators that divide planning and control tasks among *actors* or *agents* to simultaneously analyze process outputs, evaluate and judge failures, anomalies, or inefficiencies, maintain registers of and integrate new subsystems and resources, predict points of failure, and manage data organization and safety functions.^68^ Biomedical system-of-systems frameworks have so far been limited in implementation, with examples including modeling heterogeneous cell culture,^69^ health care management^70^ and the interaction of medical devices with human physiology,^71^ thus focusing on small-scale, individual-level challenges of multimodal data and systems. Larger-scale systems, such as multiarm drug discovery pipelines, will break new ground for these central-control autonomous systems implemented at larger scale. This increase in scale will no doubt exaggerate the challenges inherent to coordination of complex workflows that limit current task hand-off efficiency, as we now discuss.

### 3.2 Contemporary Examples of Task Hand-Off Inefficiencies

Over a decade has passed since research teams showed the potential of integrated, automated end-to-end systems and began assembling large multistage segments of the pipeline.^29,32,51,72–75^ However, within the pharmaceutical industry today, these individual stages are largely disconnected and siloed, each led by distinct teams. The result is a cobbled-together patchwork of different tools created by different companies, each utilizing and generating different formats and types of data, as illustrated by the following examples.

#### 3.2.1 Hit Expansion in Small Molecule Drug Discovery

After identifying hit compounds, scientists need to identify additional active molecules among its analogs to evaluate potential drug candidates. Cheminformatics software tools search internal compound collections and external vendor catalogs for analogs. Multiple computational chemistry software tools then prioritize those analogs for wet-lab testing based on predicted affinity, potency, and drug-like properties. Compounds are then ordered with a procurement software system. Generative AI tools may be used to create custom analogue designs for specific hypotheses, which often require bespoke synthesis and AI-based retrosynthesis planning software tools. After the analogs are received or synthesized, an assay request system orders the testing of those compounds in selected assays, with data retrieval and analysis in yet another software tool.

An individual scientist often lacks the know-how to utilize all of the aforementioned software tools, requiring assistance from colleagues across functions, further delaying the discovery process.

#### 3.2.2 Sample Registration and Tracking for Multiple Modalities

Modern drug discovery leverages a broad spectrum of therapeutic modalities such as small molecules, biologics, RNAs, and their conjugates. Traditional laboratory information management system (LIMS) solutions were developed for single modalities. Even with efforts from multiple vendors in recent years, considerable gaps still exist in all of the current commercial solutions in supporting multiple modalities. Consequently, different modalities are registered and tracked in different LIMS solutions, creating not only data silos but also significant challenges in the management of hybrid or conjugate modalities.

## 4 The Role of LLMs and Advanced Reasoning

### 4.1 Capabilities of LLMs

Only in the last few years has the advent of LLMs and advanced reasoning AI platforms allowed for effective integration of different data types and autonomous, adaptive control programming responsive to natural language input. While mostly limited to domain-specific tasks and relatively narrow use-cases, transfer learning approaches and advances in agentic AI systems have begun to unlock the potential of multisystem control and integration along the drug discovery pipeline.^76–83^ Transfer and other deep learning techniques have shown superior predictive power for pharmacokinetic properties of novel drugs,^84,85^ QSAR modeling for low-data or challenging datasets,^86,87^ and de novo drug design in low-data settings,^88^ with real-world success demonstrated in generating novel and nanomolar-potent agonists for the poorly characterized *Nurr1* orphan receptor.^89^ But, agentic AI has so far only achieved the lowest level of advanced reasoning-based autonomous planning and execution of biomedical research, limited to pre-defined tasks and methodologies.^82^

While mostly limited to domain-specific tasks and relatively narrow use-cases, transfer learning approaches and advances in agentic AI systems have begun to unlock the potential of multi-system control and integration along the drug discovery pipeline.

Advanced reasoning capabilities have started to emerge in current-generation LLM models, going beyond surface-level tokenized pattern recognition and performing deeper cognitive tasks like logical inference, planning, multistep problem-solving, and causal reasoning.^90–92^ These tasks let models chain together multiple steps, use external tools, and, perhaps most importantly, plan and revise actions dynamically. The DrugPilot LLM-based AI agent framework was recently shown to autonomously support the entire drug discovery pipeline, integrating multimodal data and efficiently coordinating the appropriate tool use for drug-response and molecular property prediction,^93^ and AgentD can autonomously retrieve biomedical data from external databases, generate drug molecule structures, predict properties, iteratively improve drug-likeness, and predict 3D protein-ligand conformations.^94^ The complex interactions, interdependencies, and trade-offs between molecular properties, as well as the ability for drugs in violation of traditional design rules, such as Lipinski’s Rule of Five,^95,96^ to achieve success, though, suggest these LLM-based tools need to be more comprehensive in their training sets or less strict in their interpretation of drug design “rules”.^93,94^

Directly planning, executing, and analyzing chemical and biological experiments, such as massively parallel screens or targeted synthesis, to augment pregenerated data, however, can further inform and validate drug molecule design and selection. Proofs-of-concept for such integrated machine control of experimental loops have taken the form of the ChemAgents^97^ and Synbot^98^ systems. ChemAgents uses an LLM-based multiagent architecture to sequentially review literature, design experiments, robotically execute laboratory tasks, and computationally analyze results in response to plain-language prompts to run an experiment. Synbot plans and executes retrosynthesis and robotics synthesis of user-provided compound structures. Both systems are limited in their generalizability, with both requiring detailed prompts to sufficiently instruct the systems to reach a prespecified goal, but they show promise in the ability to integrate chemical analysis in-line with their process and in generating and changing robot commands tailored to the task at hand in response to continuous plan re-evaluation and data collection.

These integrated experimental systems have so far been limited to synthesizing and testing small-molecule compounds,^99^ not yet capable of more complex therapeutic compounds, such as biologics, cellular therapies, or large, multidomain molecules like PROTACs or bi-/tri-specific engagers, and more advanced testing environments, such as 3D matrix or organoid cultures.^100,101^ Automated biologic production has so far been only implemented as a small-scale proof-of-concept design,^102^ and the current generation of laboratory-scale small-molecule automation systems are not amenable to complex synthetic routes or reagents, such as stereoselective reactions and purification, oxidation- or air-/moisture-sensitive reagents, low-temperature or high-pressure reactor requirements, crystallization, isolation of tar- or waxy-like intermediates, and mid-production purification or quantification steps. Together, these limitations leave a wide gap between the current capabilities of automated molecular generation and ideal open-ended and iterative therapeutic modality frameworks.

Nonetheless, the advent and more widespread implementation of LLMs across the sciences, particularly with nascent advanced reasoning models, will lead to control algorithms based on plain human language, making the implementation of similar control systems to any intended process much simpler and more accessible.

### 4.2 Multi-Agent Systems and DORA

The advent of AI research assistants and scientists, such as DORA^103–105^ or Google’s Co-Scientist,^106^ have enabled an even greater expansion of AI capabilities, now leveraging multi-agent research to generate new hypotheses and research workflows based on researcher input and existing data structures. AI scientist assistants can scan published research articles, omics datasets, and biomedical databases to suggest novel targets or pathways, linking nonobvious connections between diseases, genes, and clinical features. By analyzing experimental results and considering how they impact the hypothesis, AI scientists can synthesize high-confidence biological insights or plan the experimental testing of a revised hypothesis. Although recent additions to the biomedical research toolkit, early use-cases have already leveraged these research assistant platforms to repurpose epigenetic modifier drugs to treat fibrosis,^107^ integrate omics data and medical literature to guide precision medicine research,^108^ curate literature for enzyme kinetic data extraction,^109^ and even plan experimental workflows for mass spectrometry datasets for astrobiology.^110^ Agentic AI-based experimental orchestrators are thus already poised to enter larger-scale discovery workflows.

Design, synthesis, and validation of targeted drug molecules complete the preclinical closed-loop cycle, again taking advantage of AI platforms’ ability to scan previously underexplored corners of chemical space to achieve optimal drug design. Notably, ChemCrow^81^ and Coscientist,^80^ LLM-based platforms with Retrieval-Augmented Generation (RAG)-like abilities to utilize external tools for subtasks such as literature search, molecular property analysis and prediction, synthesis planning, and safety assessment, can take a plain-language prompt, such as “Plan and execute the synthesis of an insect repellent,” and synthesize an appropriate compound using laboratory equipment. The Language-based Intelligent Drug Discovery Agent (LIDDIA) agentic framework takes process automation a step further by integrating structural optimizations and docking analyses, mirroring traditional medicinal chemistry methods for synthesis of novel targeted drugs.^111^

However, these AI-automated chemical or drug synthesis workflows have been limited in advanced reasoning capabilities necessary for biological discovery, hypothesis generation, and iterative testing and model refinement to generate novel chemistry. Inherent to agentic AI platforms, overconfidence, oversensitivity to query formulation and compounding errors, reproducibility, and ethical governance and oversight limit the autonomy which current platforms can handle.^82^ While these systems demonstrate impressive capabilities in autonomous literature search, synthesis planning, or compound selection, they are typically constrained to narrow domains, rely on predefined task boundaries, and often function within simulation or single-modality environments. Co-Scientist, for example, is currently oriented toward hypothesis generation and experimental planning based on omics or literature data, but it lacks native integration with physical synthesis platforms or clinical prediction modules. Similarly, ChemCrow and LIDDIA implement modular LLM-based tool usage via RAG but are focused on early-phase tasks such as property prediction and retrosynthesis and do not yet orchestrate full-cycle drug programs across biological, chemical, and clinical domains. To this end, integration of clinical trial design and predictive models such as InClinico,^32^ PROCTOR,^31^ HINT,^112^ and others^113,114^ into the system architecture would allow for early alignment with regulatory pathways, a stage largely absent from current agentic AI research tools. Importantly, however, these clinical prediction platforms have yet to be externally validated in real-world settings to actually guide the design of clinical trials, perhaps due in part to the reluctance of stakeholders to embrace novel technologies in clinical trials.^115^ Before these tools can be adopted broadly into standard clinical-stage drug discovery workflows, inclusion of parallel “AI arms” alongside traditional study and control arms would help validate these models and assess their effectiveness in improving trial design.^116^

Integrating more advanced AI controllers, themselves showing signs of near-readiness for implementation in the form of the previously described research assistant agentic systems, with these synthesis/testing systems that interact with the physical infrastructure of research has the potential to further increase the speed and success rates of early drug discovery programs by shifting more reasoning and decision-making steps from human researchers onto AI platforms. Chaining together discrete and branching steps of the drug discovery pipeline in a closed-loop, automated system to reduce downtime and human-introduced biases depends on AI control algorithms running specialized AI agents in parallel, enabling continuous feedback and model refinement across the full development cycle to themselves control and communicate with the hardware and software interfaces of legacy and AI-optimized laboratory devices (Figure 2).

**Figure 2:**
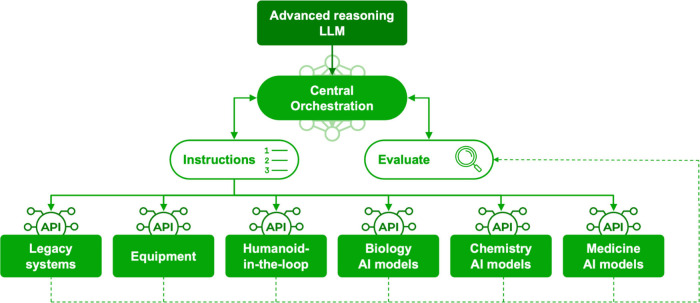
Task-specific control of physical and computational modeling systems within the drug discovery workflow. Advanced reasoning AI models centrally orchestrate individual systems with constant monitoring and fine-tuning based on data readouts and simultaneous evaluation. Each system interacts with the central orchestration model via APIs, which controls legacy biology- and chemistry-centered experimentation systems (e.g., drug screening platforms) as an equal module to predictive computational modeling of biological, chemical, or clinical systems.

Even reporting and publishing data are now falling under the domain of these automated tools, as multi-agent tools like DORA have been developed specifically to generate scientific reports.^104,105,117^ With dedicated agents for various tasks in the writing process, the full suite of humans involved in scientific writing and communication is reproduced to develop high-quality human- and machine-readable publications.

### 4.3 Limitations of LLMs and Lessons from Real-World AI Deployment

While the promise of LLM-driven and agentic AI systems in drug discovery is significant, it is essential to acknowledge the key limitations that currently constrain broader adoption. One major challenge is hallucination or the generation of confident but incorrect or unsubstantiated outputs. This is particularly problematic in biomedical applications, where fabricated citations, protein interactions, or synthetic routes can mislead downstream processes.^118^ For example, recent evaluations of LLM-generated biomedical content found high rates of hallucination when models were prompted, without purpose-made optimization strategies, to generate textual descriptions of chemical structures,^119^ propose drugs applicable for repurposing to treat Alzheimer’s disease,^120^ predict a target’s topological surface area,^121^ predict a drug’s target,^122^ interpret omics data,^123^ or identify disease-associated genes.^124^ In our own experience using early-stage agentic tools during exploratory phases of drug discovery, we observed instances in which generative models proposed molecular scaffolds that were synthetically infeasible or misaligned with known structure-activity relationships, requiring manual intervention and expert filtering, as well as instances in which manual SAR optimizations improved specific properties, such as solubility or susceptibility to metabolism.^125^

Another persistent issue is agent coordination, enabling multiple specialized agents (e.g., for chemistry, biology, and clinical trial design) to collaborate without task overlap, error propagation, or context loss. While frameworks like ChemCrow^81^ and Coscientist^80^ demonstrate tool-use capabilities, they are often brittle when agents must hand off partial results or dynamically adjust plans, underscoring the need for robust central orchestrators and agent communication protocols, which remain underdeveloped in most current implementations.

A critical challenge in multi-agent AI systems for drug discovery is the potential for cascading errors, where inaccuracies or differences from training datasets in early-stage modules, such as target pocket and activity predictions, propagate through subsequent stages, such as molecule generation, synthesis planning, and trial simulation. Even gold-standard tools like the protein structure prediction algorithm AlphaFold2 cannot be relied upon to deliver accurate predictions in every circumstance,^126^ which may pose problems when early steps in generative chemistry and virtual screening involve structure-based fitting of molecules into binding pockets and docking simulations. Such failure chains can compound if not explicitly managed through architectural and procedural safeguards. Error-avoidance and correction strategies can be borrowed from existing AI-based workflows in other areas. Interagent validation and voting apply ensemble or consensus-based decision mechanisms with redundancy;^127,128^ confidence propagation allows downstream modules to adjust their behavior accordingly;^129–131^ real-time adaptability, backtracking, and task restarts are triggered by the orchestrator when inconsistent or low-confidence outputs are detected;^132–134^ and human-in-the-loop checkpoints for patient- and regulator-facing, high-stakes transitions.^135,136^

Finally, interpretability and traceability of model outputs remain open concerns, particularly for regulatory-facing tasks. Unlike conventional QSAR or rule-based systems, LLMs often function as opaque black boxes, making it difficult to rationalize their decisions or retrace the data used for a given recommendation. This has regulatory implications for tasks such as mechanism-of-action prediction, patient stratification, and clinical trial planning, where explainability is not only scientifically desirable but legally mandated. Efforts to embed causal reasoning, evidence chains, and interpretability benchmarks into generative systems are underway^55,61,81,111,137^ but have not yet reached the reliability required for unsupervised use in high-stakes settings. Relatedly, the source, timing, and licensing status of data used to train foundation models are often undocumented, creating challenges in reproducibility, traceability, and compliance with data-use agreements and regulatory requirements in pharmaceutical pipelines.^138^

## 5 A Vision for AI-Orchestrated Drug Discovery: From Prompt to Drug

### 5.1 Conceptual Workflow

The grand objective for those developing integrated systems for AI in drug discovery is a truly prompt-to-drug autonomous pipeline—a single, plain-language request for a drug with any number of specified properties returns a synthesized, experimentally validated drug candidate ready for clinical study, along with clinical study and post-approval monitoring plans. The stages necessary to this workflow are each, individually, already actively in development for AI-driven automated control, are fully realized as commercially available products, or have been integrated into existing pharmaceutical discovery pipelines. The time is ripe for closed-loop preclinical experimental laboratories to be built from the ground up in anticipation of handing the reins to advanced reasoning AI systems for hands-off drug discovery research.

The grand objective for those developing integrated systems for AI in drug discovery is a truly prompt-to-drug autonomous pipeline—a single, plain-language request for a drug with any number of specified properties returns a synthesized, experimentally validated drug candidate ready for clinical study, along with clinical study and post-approval monitoring plans.

### 5.2 Detailed Workflow Overview

Prompting the system would begin with a simple request, such as “Design a drug for idiopathic pulmonary fibrosis (IPF).” An advanced reasoning AI model would be the director of the rest of the operation, with target discovery, chemistry, and clinical development subsystems granted the freedom to identify optimal targets, molecular design, and patient populations, respectively, to increase the likelihood of success (Figure 3). Spawning a research plan and teams of AI agents, the model has learned how to learn, integrating publicly available and privately owned data with published literature to set in motion a centralized drug discovery program, similar to autonomous experimental systems that have shown promise in centrally organizing stepwise, agent-driven experimental plans, such as ChemCrow^81^ or ChemAgents.^97^ Cross-checking the research plan with competitive analyses—also used to constantly reassess and realign the program after each key data readout—is key to maximize the chance of commercial success.^139,140^

**Figure 3:**
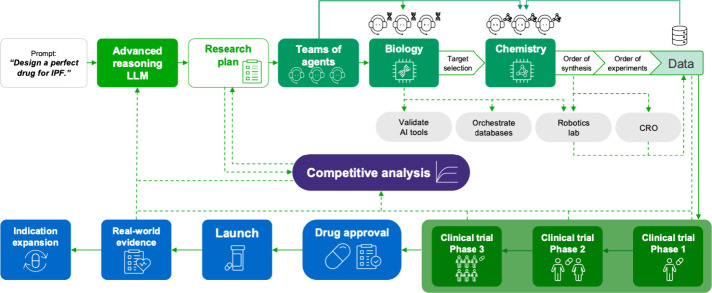
Theoretical optimized workflow for autonomous drug discovery with minimal researcher input. A controller advanced reasoning model plans and executes a research plan comprised of hands-off *in vitro* and *in silico*-based target discovery, molecular discovery, and chemical analysis. The resulting candidate drug molecules and supportive preclinical data inform clinical trial design. Clinical trial readouts and postapproval evidence continually feed the competitive analysis module, in turn refining the advanced reasoning model and subsequent research plan to inform drug development based on the greater drug development landscape (i.e., competitor drugs, regulatory considerations, research and funding trends, unmet patient needs, etc.).

Biology agents operate automated laboratories, nominating disease-associated targets from compiling published data sources and *in vitro* experimental validation models (Figure 3). These discovery-based modules scan the literature, devise hypotheses and experimental plans, and validate as necessary with *in vitro* experimentation, as shown in early protypes of controllers like DORA and Coscientist,^80,103^ or in more specialized laboratory experiment planners like CRISPR-GPT.^141^ With a target in hand, the chemistry agents use proprietary^74^ or publicly available generative chemistry platforms^142^ to design targeted drug molecules. The chemistry module follows the traditional stepwise molecular lead optimization task chain using established and validated docking models, synthetic accessibility scoring, and ADMET prediction algorithms to prioritize the most promising lead molecules.

While LLMs excel at symbolic reasoning and task coordination, they lack deep biochemical and structural grounding, which demands that physics-based models such as molecular dynamics (MD), quantum mechanical (QM) simulations, and docking engines be integrated into LLM-driven workflows. Beyond strictly LLM-based methods relying solely on SMILES or SELFIES encodings, next-generation models may incorporate 3D molecular graphs, electron density maps, and experimental assay data into a unified latent space trained on multimodal foundation models. Agents could propose candidates and then perform external simulations to validate binding affinity, conformational stability, or synthetic feasibility. In our experience, such hybrid approaches were instrumental in refining AI-generated candidates during the design of the novel TNIK inhibitor rentosertib, in which the Chemistry42 platform leverages a variety of 2D and 3D structural models to identify the most promising lead molecules. Looking ahead, modular architectures that combine language-based planning with simulation and multimodal data will be critical for advancing accuracy, interpretability, and real-world applicability.

A limitation inherent to AI discovery frameworks is the difficulty in distinguishing correlation from causation, as the pattern recognition modules, as discussed, lack biochemical grounding or human-like understanding.^82^ Relatedly, AI tools often have a blind spot for context, with cell type-dependent expression or function and pleiotropic effects giving genes complex roles that, if not explicitly captured by training datasets, may be completely overlooked in target discovery efforts.^143^ These shortcomings inevitably require follow-up validation with in-house systems or CROs conducting synthesis, *in vitro*/*in vivo* testing, and molecular optimizations. AI-based molecular design tools combining minimal-step synthesis planning^144^ with on-chip microfluidic synthesis^145^ and robotics-integrated cellular assays^99^ show that simplified synthesis schemes enabling rapid manufacture, testing, and scale-up of novel lead compounds are easily obtainable with today’s automation tools.

The intersection of these predictive and generative tools with the complex and dynamic nature of biological networks and individual molecules necessitates validation of any model with physical experimentation, and often iterative optimization, before any drug can enter clinical, or even pre-clinical, testing.^126,146^ Recent developments in integrating design–make–test–analyze (DMTA) frameworks into drug and materials discovery efforts illustrate how iterative model refinement based on closed-loop automated cycles of the synthesis, testing, and optimization of AI-discovered compounds leads to robust drug discovery systems and self-reinforcing improvement of the datasets used to guide molecular generation.^83,97,98,147–150^

The biological insights and preclinical testing data generated at these stages inform subsequent clinical trial designs, in which clinical prediction models, such as the recently developed PROCTOR,^31^ InClinico,^32^ or HINT^112^ models, prospectively identify patient populations and clinical trial designs most likely to achieve success in clinical testing.

AI systems have not, however, replaced the gold-standard multistage, progressive clinical trials for assessing safety, setting optimal dosage, and evaluating efficacy of novel drugs. It is therefore imperative to align with government regulators on the capabilities of validated AI systems, as they remain the authoritative gatekeepers of clinical trial progression, and the importance of their confidence in the proficiency of these systems can therefore not be understated.

Phase IV and head-to-head clinical trials act as a constant source of additional real-world data, perhaps even richer than phase I–III trials (Figure 3). With a more diverse and larger patient population than the limited clinical trials and with longer postadministration follow-up, the biology, chemistry, clinical development, and competitive landscape models are constantly updated and refined to inform future drug discovery efforts.

Such a workflow does not need to arise from scratch. As mentioned, individual module-specific AI models exist or are in active development. What lies ahead is the successful validation of single multimodal, multi-omic, highly capable models trained on the output from these experimentally validated limited models to facilitate the transition toward such a Pharmaceutical Superintelligence (PSI).

### 5.3 The Role of API-Driven and Humanoid Agents

The past century of traditional drug discovery has standardized many important steps throughout the process. In addition to next-generation AI models for biology, chemistry, and clinical development, legacy experimental and data analysis systems should remain integral to the drug development pipeline. For example, single-cell transcriptomic assays,^151^ by now easily automated and highly parallelized,^152^ are the gold-standard for assessing dynamic phenotypic effects across cell types. Today’s high-throughput chemical synthesis and analysis workflows, as well, enable the massive screens required to train AI models and for hyperefficient synthesis-test-refine cycles.^153–157^

Nonetheless, the human interaction with the system should be minimized to avoid errors, biases, and downtime. Inherent to the design of humanoid robots is the ability to control legacy equipment and workspaces designed for use by human scientists. Humanoid robots would function simply as one agent deployed by the central advanced reasoning AI. Able to work in extended, uninterrupted shifts and with the ability to seamlessly hand off tasks to other capable robots, a humanoid-in-the-loop would minimize the between-steps downtime within and between the highly technical stages of biological and chemical experimentation. Insilico Medicine, for example, is already developing humanoid-in-the-loop workflows to complement their autonomous preclinical laboratory facilities that have already yielded insights into anti-aging/senomorphic therapeutics.^99^

## 6 Future Outlook and Recommendations

### 6.1 Toward Multimodal and Program-Specific Models

Only when all available data are taken into account in the biological, chemical, and clinical design of a novel drug program can we hope to achieve the maximal likelihood of success. Everything from spatially resolved multiomics data sets and anonymized patient medical records, to binding free energy and docking models, to clinical trial participant omics and matched outcomes, all will be necessary to train the multimodal AI platforms of the future.

In balance with acquiring the most data possible, the AI models will simultaneously need to be tailored to each drug discovery program, with an eye toward filtering the data on which the model is trained. For example, if the goal is to therapeutically target dysfunctional processes in a highly sensitive and important cell type, maybe a neuron, care must be taken, perhaps more than is typical, to reduce cytotoxic or off-target effects, which would require filtering of target training datasets to preclude such possibilities or specifically to account for brain-penetrance and reduced efflux. The multimodal training data for each program should therefore be only as wide as is necessary yet as deep as possible.

### 6.2 The Role of Human Oversight and Accountability in Closed-Loop Autonomous Drug Discovery

While the future we envision for accelerated drug discovery relies on the autonomous execution of the drug discovery pipeline, we recognize that accountability, safety assurance, and legal responsibility cannot themselves be transferred to AI systems. Therefore, safe and responsible deployment of autonomous drug discovery frameworks requires guiding principles and checks grounded in equitability, patient-centricity, and fairness.

The fallibility of AI models, as exemplified by LLM hallucinations, requires careful oversight to ensure accurate and safe results. All outputs should be accompanied by machine-readable records of the software and hardware versions, input data, and reasoning steps used to generate decisions, molecular designs, and synthesized compounds, ensuring auditability and reproducibility. The ability for LLMs to recognize errors and issue feedback on their own responses or to initiate self-correction is still challenging in most situations, making it so that full system autonomy will require further progress in self-correction capabilities before such systems can be trusted with minimal human intervention.^158^ At least in near-term implementations, systems should therefore include mechanisms for human operators to evaluate, pause, modify, or veto AI-driven decisions, especially in stages with patient-facing implications.

A key player in the drug discovery ecosystem is the collection of legal and regulatory bodies with a purview over drug discovery processes worldwide, and these bodies will both require input from those in the field in order to devise appropriate and fair guidelines for such a nascent process and enforce such guidelines to govern those in the field. Developers of autonomous drug discovery workflows must maintain close engagement with regulatory bodies to develop standards for validation and approval of AI-generated trial designs, biomarker strategies, and patient stratification plans. A major checkpoint for the implementation of such systems is likely to be sufficient evidence of safe and effective AI tool performance within each step of the process individually before any product of an autonomous pipeline reaches humans in the clinic. Simultaneous and downstream of regulatory oversight, AI systems must be subject to the same ethical review processes that apply to human-generated research with active monitoring for population biases in training data and outcome recommendations. All AI tools accessing patient-level, proprietary, or regulated data should operate under strict compliance with data privacy frameworks, such as HIPAA, GDPR, and institutional governance protocols. Training and fine-tuning should be performed on deidentified, legally cleared datasets.

### 6.3 Recommendations for the Field

These monumental advances in AI-driven drug development just over the horizon cannot be reached by any single entity, not by an academic research group, not by an AI-focused biotechnology company, and not by big pharma. Achieving truly end-to-end, autonomous drug development will require buy-in from the entire sector, with each player contributing a necessary piece of the puzzle.

We recommend that research groups publish their work at every stage. Academic journals provide not only easily mined text and data to train biological network and language models on biochemical interactions but also a vetted, peer-reviewed outlet for instilling confidence in the soundness of the science underlying these advances. Incremental and breakthrough developments in AI-based drug discovery should be shared broadly and immediately for maximum impact. Insilico Medicine has prioritized publication of the findings throughout the drug development process, as exemplified by manuscripts comprehensively describing the target discovery,^159^ molecular design,^125^ and clinical stages of rentosertib (ISM001-055) development.^2,159^

To keep AI-based workflows future-friendly, we recommend research groups fully annotate their AI models validated for specific tasks, treat each automated closed-loop subsystem as equal pieces of lab equipment, and design their protocols from the very start to operate their subsystems via APIs that central-orchestration AIs can access and direct. While each individual subsystem can and should be tailored to each drug development program, siloed from the rest of the processes, the interoperability and implementation of the platforms across research groups depend on the usability by central control models and the interpretable architecture and instructions they can leverage.

While the long-term goal of AI-orchestrated drug discovery is to reduce human error and bias, we acknowledge that removing human oversight entirely is neither advisible nor feasible under current scientific, legal, or regulatory conditions. Instead, the optimal approach should include human-in-the-loop checks to ensure safety, transparency, and public trust: model provenance and traceability, human override capability, regulatory alignment, ethical review, and bias monitoring and data security and compliance. Prior to adoption in human-facing therapeutic development, each individual step controlled by a central AI would need validation in a sufficiently large set of approved drugs so that the public and regulators can be sure that AI tools have the capability to deliver safe and effective therapies. Although presenting a significant bottleneck to the implementation of end-to-end AI-based drug discovery programs, these subsystem validations are crucial for getting all stakeholders on board. Early successes that validate aspects of the AI-centric approach include the advance of rentosertib through early phases of clinical testing, demonstrating that AI tools can identify biologically informed disease targets and design safe and effective drug molecules. However, other subsystems, such as clinical trial design modules, need novel validation methods, such as parallel “AI arms” in clinical trials, to reach a similar level of real-world evidence.^116^

## 7 Conclusion

The integration of AI into the drug discovery pipeline is rapidly transforming the pharmaceutical industry, shifting the paradigm from fragmented, manual processes to autonomous, data-driven workflows.

As large language models and advanced reasoning systems mature, their ability to orchestrate end-to-end discovery, from hypothesis generation to molecular synthesis, biological testing, and clinical planning, continues to evolve and is capable of adapting dynamically to real-time data and guiding programs across multidisciplinary domains.

The integration of AI into the drug discovery pipeline is rapidly transforming the pharmaceutical industry, shifting the paradigm from fragmented, manual processes to autonomous, data-driven workflows.

The realization of a true “prompt-to-drug” pipeline, in which a natural language request initiates a fully autonomous drug development program, is no longer a distant aspiration. With the development of modular AI platforms, humanoid-in-the-loop robotics, and multi-agent systems, the foundational components for this vision are already operational. When combined with advanced generative models, program-specific training, and closed-loop experimental laboratories, these systems not only reduce development timelines and costs but also unlock new levels of scientific discovery by minimizing human bias and enabling the exploration of previously underexplored areas of chemical and biological space.

However, to bring this vision to full fruition, collaboration across academia, biotechnology companies, and regulatory agencies is imperative. Efforts must be directed toward ensuring data interoperability, transparent model reporting, and alignment with regulatory standards to build trust in AI-assisted decision-making. By continuing to publish results, share data, and standardize interfaces for AI-human collaboration, the field can move toward a future where AI is not just a tool but a co-scientist, driving innovation, improving patient outcomes, and transforming how we develop medicine.
